# Molecular and Cellular Mechanisms of Static and Repetitive Magnetic Stimulation in Cancer Therapy: A Scoping Review

**DOI:** 10.3390/biomedicines14030638

**Published:** 2026-03-12

**Authors:** Dirson Stein, Amanda Stieven, Rodrigo Hernandes Paludo, Khetrüin Jordana Fiuza, Lucas Rosa Fraga, Felipe Fregni, Wolnei Caumo, Mariane da Cunha Jaeger, Iraci L. S. Torres

**Affiliations:** 1 Laboratory of Pain Pharmacology and Neuromodulation: Preclinical Investigations, Hospital de Clínicas de Porto Alegre (HCPA), Porto Alegre 90035-003, Brazil; dirstein@gmail.com (D.S.); rpaludo@hcpa.edu.br (R.H.P.); khetruinfiuza@hotmail.com (K.J.F.); 2 Postgraduate Program in Medicine: Medical Sciences, Department of Medicine, Universidade Federal do Rio Grande do Sul (UFRGS), Porto Alegre 90035-903, Brazil; amandastieven13@gmail.com (A.S.); lrfraga@hcpa.edu.br (L.R.F.); wcaumo@hcpa.edu.br (W.C.); 3 Laboratory of Neuromodulation and Center for Clinical Research Learning, Physics and Rehabilitation Department, Spaulding Rehabilitation Hospital, Boston, MA 02129, USA; fregni.felipe@mgh.harvard.edu; 4 Children’s Cancer Institute, Porto Alegre 90620-110, Brazil; labpesquisa1@ici.ong

**Keywords:** neuromodulation, cancer, repetitive magnetic stimulation, static magnetic stimulation, preclinical, in vitro, in vivo

## Abstract

Repetitive magnetic stimulation (rMS) and static magnetic stimulation (sMS) are currently employed as adjunctive therapies for specific neurological conditions. Despite substantial advances in cancer treatment, unfavorable prognoses and outcomes persist, especially for aggressive tumors, including glioblastoma and acute myeloid leukemia. The utilization of magnetic fields has shown antitumoral benefits in both in vitro and animal studies, suggesting its potential as an efficient non-invasive therapeutic approach; nevertheless, the precise mechanisms of action remain unclear. This scoping review intended to identify published research investigating the effects of sMS and rMS in in vitro and in vivo models to evaluate their impacts on morphological and molecular parameters. Four databases (PubMed, Embase, Web of Science, and Scopus) were assessed; the search strategy was limited to the past twenty-five years of data publication. Studies utilizing rMS or sMS as a treatment for conditions other than cancers, as well as those not considering these therapies as adjunctive therapy, were eliminated. Nine articles using rMS were included: three in vitro, two employing animal models, and the remaining four including both cellular and animal-based analyses. Sixteen studies using sMS were identified: twelve in vitro, three in vivo, and one with both models. The findings show that both rMS and sMS elicit a diverse array of biological responses in cancer cells, which are very variable and greatly influenced by tumor type, stimulation frequency, magnetic field intensity, exposure length, and experimental conditions.

## 1. Introduction

Non-invasive neuromodulatory approaches, including those employing magnetic and electric fields, have markedly progressed the domain of nonpharmacological disease therapy [1]. In recent years, extensive preclinical and clinical research has examined the application of neuromodulatory techniques for several conditions, advancing the understanding of their use in neurological conditions like epilepsy [2], Parkinson’s disease [3], major depression [4], anxiety [5,6], chronic pain [7], and cancer-related pain [8,9], among others [10,11]. These are interventions aimed at modulating brain activity by stimulating specific neural networks, especially promising for cases resistant to pharmacological agents [12].

Among several non-invasive brain stimulation (NIBS) methods, transcranial magnetic stimulation (TMS) is acknowledged for its usefulness, being widely used in primary clinical neuroscience [13]. The first documented application of TMS took place in 1993 for drug-resistant major depression [14]. Currently, TMS is being used for Alzheimer’s disease, stroke, multiple sclerosis, pain, anxiety disorders, and substance abuse [11,15]. Furthermore, a pilot study has suggested that repetitive TMS (rTMS) is a safe and effective therapeutic approach for improving peripheral nerve damage and relieving the symptoms of chemotherapy-induced peripheral neuropathy in patients with multiple myeloma [16]. The method requires an apparatus equipped with a high-current pulse generator that produces discharge currents, which flow through a stimulating coil to generate a brief magnetic pulse with field strengths reaching several Tesla, generating secondary electric fields [13,17]. Upon placing the coil on the subject’s head, the magnetic field suffers minimal attenuation by extracerebral tissues, generating an electric field sufficiently strong to depolarize superficial axons and stimulate neuronal networks [18]. TMS can be used for diagnostic and therapeutic purposes for numerous neurological disorders, non-invasively examining cortical excitability, connectivity, and motor processes [13]. According to the purpose, it is performed in many protocol patterns and encompasses a wide variety of intensities, frequencies, and durations. While single and paired pulses are used for diagnostic purposes, rTMS is mostly aimed at treating neurological diseases [13,19].

Studies demonstrate that rTMS at frequencies over 3 Hz enhances neuronal activity, whereas rTMS at lower frequencies (below 1 Hz) typically reduces synaptic efficacy [20]. Additionally, rTMS can be administered using a method referred to as theta burst stimulation (TBS), characterized by magnetic pulses in bursts of three, at high frequency (50 Hz), with an interburst interval of 200 ms (5 Hz, which is in the range of theta frequency). TBS can be configured for continuous (cTBS), which primarily causes inhibitory effects via long-term depression (LTD), or intermittent (iTBS), which applies bursts with pauses, leading to excitatory effects via long-term potentiation (LTP) [10]. In contrast, static magnetic stimulation (sMS), unlike other stimulation modalities, does not induce electric currents [21]. It employs neodymium magnets, which can be positioned on the subject’s scalp, that generate static magnetic fields that primarily affect the synapse, changing the properties of membrane ion channels, influencing neuronal excitability, and potentially resulting in enduring changes in neuronal plasticity [21,22]. In addition to recent research employing repetitive magnetic stimulation (rMS) [23], emerging studies using sMS, including two conducted in our research group, have demonstrated its capacity to inhibit the migration and telomerase function in vivo, diminish the survival and viability of neuroblastoma cells in vitro, and reduce cisplatin resistance by enhancing apoptosis pathways and genotoxicity in cancer cell lines, exhibiting significant antineoplastic effects [24,25,26,27,28].

Cancer is one of the leading causes of death worldwide, with a growing trend resulting from population growth, increased life expectancy, and the widespread adoption of high-risk lifestyles [29,30,31]. The disease arises from genetic alterations that lead to uncontrolled cell proliferation and metastasis. Current treatment strategies involve different approaches used alone or in combination, such as surgery, radiotherapy, chemotherapy, immunotherapy, and targeted therapies [32,33]. Despite significant advances in cancer treatment over recent decades, prognosis remains poor for aggressive malignancies such as glioblastoma and acute myeloid leukemia. Factors including tumor heterogeneity, treatment resistance, and patient-specific characteristics continue to contribute to unfavorable clinical outcomes, even with the development of novel therapies [34]. Moreover, cancer treatment can induce several side effects, including fatigue, nausea, alopecia, neuropathies, and chronic pain, as well as oral lesions, cognitive impairments, mental health, and appetite, gastrointestinal, dermatological, fertility, and sexual alterations [35].

In light of the lack of effectiveness and adverse effects of the current antitumoral therapies, researchers must explore novel approaches, especially as a complement for cancer management (for a review, see [36]). While the use of magnetic fields has exhibited remarkable antitumoral effects in both in vitro and in vivo experimental studies, suggesting it as an effective non-invasive therapeutic approach [28,37,38], the exact underlying mechanisms of action remain unclear. Therefore, this scoping review intended to identify research investigating the effects of sMS and rMS in both in vitro and in vivo trials to evaluate protocols and their impacts on morphological and molecular parameters.

## 2. Relevant Sections

### 2.1. Terminology

The acronyms sMS, sMF, PEMF (or pulsed MF), rMS, and rTMS are sometimes used interchangeably; however, they refer to conceptually and technically distinct neuromodulation approaches. To avoid conceptual overlap and to guarantee appropriate interpretation and comparison of neuromodulation studies, it is imperative to make these distinctions clear.

Static magnetic stimulation (sMS) or static magnetic field (sMF) refers to the application of a constant (non-time-varying) magnetic field, typically using permanent magnets, which does not induce electric currents but is hypothesized to modulate neuronal activity through different biophysical mechanisms (e.g., effects on ion channels or membrane properties). In turn, the Pulsed Electromagnetic Field (PEMF) is a broad, generic term referring to any therapy that uses time-varying magnetic fields. The field strength is usually low to moderate, and it is typically employed in orthopedics (e.g., bone healing), pain therapy, and anti-inflammatory treatments. The devices are often portable or wearable. In many PEMF systems, the magnetic field is too weak or too slow to induce significant cortical depolarization. Repetitive magnetic stimulation (rMS) is a general term that refers to time-varying magnetic fields applied to biological tissues. It can be applied to the brain (cortical rMS) and peripheral nerves (peripheral rMS), as well as muscles or nerve roots. Thus, rMS describes the stimulation pattern (repetitive magnetic pulses) but does not specify whether the target is central or peripheral. In contrast, repetitive transcranial magnetic stimulation (rTMS) is a specific form of rMS designed to deliver focal, high-intensity magnetic pulses through the scalp and skull to induce electric currents in cortical tissue, thereby directly modulating neuronal excitability and synaptic plasticity in targeted brain regions. Thus, these techniques differ along key operational dimensions: (i) field dynamics (time-varying in PEMF/rMS/rTMS vs. static in sMS/sMF), (ii) mechanism of action (electromagnetic induction and direct modulation of cortical excitability in rTMS vs. non-inductive, putative membrane- or channel-level effects in sMS/sMF), and (iii) spatial focality and depth of action (typically focal and cortex-targeted in rTMS, broader or less focal in generic rMS and PEMF, and more diffuse/superficial in sMS/sMF) [39,40,41].

### 2.2. Protocol

This scoping review was conducted in compliance with the Preferred Reporting Items for Systematic Reviews and Meta-Analyses extension for Scoping Reviews (PRISMA-ScR) guidelines. The PRISMA-ScR framework was used to guide the methodological process, including the formulation of the research question, identification of relevant studies, selection of studies, data charting, and synthesis of findings [42] (Supplementary File S1).

### 2.3. Search Strategy

To identify the literature concerning rMS and sMS for possible adjunct treatment for cancer, four databases (PubMed, Embase, Web of Science, and Scopus) were electronically searched. We designed and implemented a search strategy within the designated databases, which included terms related to “cancer” and “repetitive magnetic stimulation” OR “static magnetic stimulation”, “in vitro”, and “rodents” OR “animal model”. Boolean operators (AND/OR) were used to combine terms appropriately for each database (Supplementary File S2).

In the assessed databases, the keyword “pulsed magnetic field” was used to denote rMS, whereas the term “magnetic field” refers to both rMS and sMS. Furthermore, the search strategy was limited to the last twenty-five years of data publication (from 2000 to June 2025) to include all studies in this field that use newer techniques and parameters for MS (magnetic stimulation) applications.

The selection of articles encompassed studies conducted in vitro using human or animal cell lines, as well as animal studies that evaluated the effects of MS on rodent species. Eligible studies must have reported on outcomes such as cell viability, gene expression, or histopathology responses in animals. Studies using other treatment methods, like chemotherapy, were also included, assuming that MS was also employed.

The exclusion criteria were established on the following basis: a full text in a language other than English, studies that were devoid of definitive outcomes or relevant interventions, studies that focused on human subjects or in silico methods, studies that did not regard rMS/sMS as an adjunctive therapy, studies with rMS or sMS as a form of treatment for conditions other than cancers, studies that used rMS/sMS to address symptoms of diseases in oncology patients, and studies that employed rMS as a surgical aid or diagnostic tool for benign and malignant tumors.

Titles and abstracts were independently screened by two reviewers. Disagreements were resolved by consensus, and a third reviewer was consulted if needed. Full-text screening was performed for eligible studies. In the process, the software Rayyan was used to organize and delete duplicate articles. The data extraction was performed using a qualitative descriptive approach to categorize the included studies based on the name of the authors, year of publication, type of study, stimulation protocol, outcome assessment, and conclusion of the intervention (Table 1 and Table 2).

### 2.4. Risk of Bias Assessment

The risk of bias of the included studies was assessed independently for in vitro and in vivo studies using validated, study design-specific instruments. In vitro studies were evaluated with the QUIN (Quality Assessment Tool for In Vitro Studies) tool [43]. In vivo studies were assessed using the SYRCLE Risk of Bias tool for animal studies [44]. Two reviewers independently conducted all risk of bias assessments, with discrepancies resolved through discussion when necessary.

**Table 1:** Studies employing repetitive magnetic stimulation (rMS) as a potential antitumoral therapy in in vitro models.

First Author (Year)	Cell Line	Stimulation Protocol Magnetic Field	Outcome	Main Results
Jo et al. (2025) [23]	U87MG-like glioblastoma human cell line TS15-88 glioblastoma cell line TS21-117 glioblastoma cell line	Customized rMS (Bicon-1000Pro, Mcube Technology, Seoul, Korea) Stimulation delivered a monophasic pulse with a rise time of 370 μs Frequency: 0.5 Hz with on–off interval of 3 s Intensity: 18 mT Duration: 3 days for 10 min/day	CCK-8 assay RNA-seq transcriptome analysis RT-qPCR ATP assay 3D invasion assay Western blot TUNEL assay Sphere formation assay	Inhibition of cell proliferation by downregulating the expression of *FLNA* and *FLNC* Suppression and sphere formation Induction of apoptosis by downregulating ERK/JNK/p38 and PI3K/AKT/mTOR pathways Activation of Bax and inhibition of Bcl-2 Prevention of invasion of glioblastoma by downregulating the expression of *MMP2* and *MMP9* Suppression of the activation of *EGFR* and *EphA2*
Ashdown et al. (2020) [45]	A549 human lung cancer cells Lewis lung carcinoma (LLC) MDA-MB-231 human breast carcinoma cells Lung lymphatic endothelial cells (control) (hLECs)	Solenoid magnet (#R-2016-12; Magnatech, Maple Grove, MN, USA) interrupted by a circuit and connected to a standard power supply (BioRad Power Pack, 10 V DC current; Bio-Rad, Hercules, CA, USA) Intensity: 20 mT Rate of rise (dB/dt) in the msec range Frequency: sequential 50 and 385 Hz oscillating MF Duration: 10 min	Cell membrane integrity assay Flow cytometry AAf-glo cytotoxicity Heparin Lyase III CellTiter-Glo assay Scanning electron microscopy	**A549, LLC, and MDA-MB-231 cells** Induced intracellular protease release Disruptive effect in the glycocalyx HS Altered membrane integrity and reducing cell viability Increased cytotoxicity Reduced cell proliferation **hLECs** No effects on membrane integrity, protease release, cell viability or cytotoxicity
Heng et al. (2022) [46]	HuH7 human hepatocellular carcinoma cells (HCC) HCT116 human colorectal adenocarcinoma cells (CRC)	Magstim Rapid2 Plus instrument (Magstim Ltd., Spring Gardens, Whitland, UK) equipped with the D70 air film coil (AFC) **iRMS:** Frequency: 1, 5, 10, 20, and 40 Hz Intensity: 0.8 T Duration: 20 min **bRMS:** Frequency: 40 Hz/100% power; 40 Hz/50% power Intensity: 0.4–0.8 T Duration: 20 min	MTT assay: cell viability	Opposite trends in how CRC and HCC cells respond to the frequency, temporal patterns, and magnetic field flux density, which were considered the parameters of the experimental rMS High frequency of pulses, burst patterns, and shorter cycles suppress the cell viability
Jo et al. (2022) [47]	Neuro-2a (N2a) cell line: neuroblast model	Customized rMS (Bicon-1000Pro, Mcube Technology, Seoul, Korea) Stimulation delivered a monophasic pulse with a rise time of 370 μs Frequency: 0.5 Hz with on–off interval of 3 s Intensity: 18 mT Duration: 3 days for 10 min/day	CCK-8 assay CFA Colony formation assay (CFA) RNA-seq transcriptome analysis RT-qPCR Western blot TUNEL assay IHC	Suppression of cell proliferation and tumor progression Reduced Ki-67 expression Decreased colony formation Decreased levels of phosphorylated *JNK*, *p38*, *ERK*, *PI3K*, *AKT*, and *mTOR* Downregulation of the Wnt/β-catenin signaling pathway Induction of apoptosis
Lee et al. (2015) [48]	Immortalized mouse neuroblastoma cell line N1E-115 Groups: ○ Control ○ Low-frequency rMS ○ High-frequency rMS	Circular coil Frequency: 0.5 Hz with on–off interval of 3 s, and 10 Hz Intensity: 1 T (100% machine output stimulation intensity) Duration: 3 days for 20 min/day	Western blot Blockage of growth factors Cell proliferation: microscopy	**High-frequency (10 Hz)** Increased cell proliferation Increased protein levels of BDNF, GDNF, NT-3, PDGF, ERK, and Akt **Low-frequency (0.5 Hz)** Decreased ERK and Akt levels
Yamaguchi et al. (2004) [49]	B16-BL6 murine melanoma cell line	Magnetic stimulator (Nihon Kohden Co., Tokyo, Japan) which delivered biphasic cosine current pulses with a period of 238 ms Intensity: 0.75 T Frequency: 25 pulses/s, and four patterns of stimulation ○ (1) 40 s (1000 pulses) ○ (2) 80 s (2000 pulses) ○ (3) 120 s (3000 pulses) per 1 day (for groups 1, 2 and 3) ○ (4) 1000 pulses/day for 3 days (3000 pulses)	MTT assay	No significant differences in cell viability between the control and stimulated groups
Yamaguchi et al. (2006) [50]	B16-BL6 murine melanoma cell line	Magnetic stimulator (Nihon Kohden Co., Tokyo, Japan) delivering biphasic cosine current pulses with a period of 238 ms Intensity: 0.25 T Frequency: 25 pulses/s, and 1000 pulses/sample/day; 80 s for 16 days Evaluations at 3 and 7 days	MTT assay: cell viability	No effect of rMS on cell viability

Caption: BDNF: brain-derived neurotrophic factor; CFA: colony formation assay, CRC: colorectal cancer; dB/dt: the rate of change of b with respect to time; GDNF: glial cell line-derived neurotrophic factor; HCC: hepatocellular carcinoma; IHC: immunohistochemistry assay; iRMS: intermittent repetitive magnetic stimulation; mT: milliTesla; MTT: 3-(4,5-dimethylthiazol-2-yl)-2,5-diphenyl-2H-tetrazolium bromide; NT-3: neurotrophin-3; PDGF: platelet-derived growth factor; RT-qPCR: reverse-transcriptase polymerase chain reaction; s: seconds; T: Tesla; TUNEL assay: terminal dUTP nick-end labeling.

**Table 2:** Studies employing repetitive magnetic stimulation (rMS) as a potential antitumoral therapy in in vivo models.

First Author (Year)	Animal Model	Stimulation Protocol Magnetic Field	Outcome	Main Results
Jo et al. (2025) [23]	Male athymic nude mice orthotopic xenograft model: U87MG TS cells implanted into the right frontal lobe by stereotaxic injection Groups: ○ Sham ○ rTMS ○ TMZ 30 mg/kg	Customized rTMS (Bicon-1000Pro, Mcube Technology, Seoul, Korea) Frequency: 0.5 Hz with on–off interval of 3 s Intensity: 18 mT—monophasic pulse with a rise time of 370 μs Duration: 21 days for 10 min/day	RT-qPCR TUNEL assay IHC H&E MRI Bioluminescence imaging	**rTMS or TMZ:** Suppression of tumor progression and tumor mass by downregulating the expression of *FLNA*, *FLNC*, and *Ki-67* Inhibited ERK/JNK/p38 and PI3K/AKT/mTOR pathways via downregulation of the EGF receptor and EphA2 activation Increased apoptosis Prolonged life span
Tatarov et al. (2011) [38]	Swiss outbred female nude mice (Cr:NIH(S)-nu/nu) injected with metastatic mouse breast tumor cell line EpH4-MEK-Bcl213	Helmholtz coil driven by a controllable high-power alternating-current supply (SDR TH 40–250, Sodilec, Bordeaux, France) Intensity: 100 mT Frequency: 1 Hz Duration: daily for 60, 180, or 360 min for 4 weeks	TUNEL assay for fragmented nuclei Tumor growth progression Histopathology	**rMS 360 min daily/4 wk:** Suppressed tumor growth Induced extensive areas of necrosis and apoptosis Did not induce histopathological damage to healthy tissues
Jo et al. (2022) [47]	BALB/c nude male mice xenograft model: Neuro-2a (N2a) neuroblastoma cells inoculated subcutaneously into the right hind limb anterior root ganglion Groups: ○ Sham ○ rMS ○ TMZ 30 mg/kg	Customized rMS (Bicon-1000Pro, Mcube Technology, Seoul, Korea) Frequency: 0.5 Hz with on–off interval of 3 s Intensity: 18 mT—monophasic pulse with a rise time of 370 μs Duration: 18 days for 10 min/day	RT-qPCR Western blot TUNEL assay IHC Tumor progression	Reduced tumor volume and progression Decreased *WNT3a*, *WNT5a*, *β-catenin*, *DVL1*, and *LEF1* gene expression Downregulated the protein expression levels of WNT3a, WNT5a, β-catenin, DVL1, LEF1, cyclin D1, and c-Myc Downregulated the Wnt/β-catenin signaling pathway Induced apoptosis
Yamaguchi et al. (2004) [49]	C57BL/6J female mice inoculated with B16-BL6 murine melanoma cell line Groups: ○ (1) Stimulated ○ (2) Sham ○ (3) Control	Magnetic stimulator (Nihon Kohden Co., Tokyo, Japan) which delivered biphasic cosine current pulses with a period of 238 ms Intensity: 0.75 T Frequency: 25 pulses/s; 1000 pulses/day Duration: 16 days	Tumor weight	No significant differences in the tumor weight between the stimulated group and the sham group
Yamaguchi et al. (2006) [50]	Female C57BL/6J mice inoculated with B16-BL6 murine melanoma cells Groups: ○ (1) Stimulated ○ (2) Sham	Magnetic stimulator (Nihon Kohden Co., Tokyo, Japan), which delivered biphasic cosine current pulses with a period of 238 ms Intensity: 0.25 T Frequency: 25 pulses/s; 1000 pulses/day Duration: 16 days	Histopathology Tumor weight Immunoassay: spleen IL-2 and TNF-α levels at days 3 and 7	**High-frequency rMS:** Suppression of tumor growth (54%) Extensive areas of necrosis Increased spleen TNF-α levels after 3 and 7 days Longer survival
Perrino et al. (2024) [51]	Male Sprague–Dawley rats injected with C6 glioma cells Groups: ○ (1) rTMS ○ (2) IGF-Trap + sham rTMS ○ (3) IGF-Trap + real rTMS Male NSG mice injected with the murine glioma GL261 cells Groups: ○ Vehicle ○ rTMS + vehicle ○ IGF-Trap alone ○ rTMS + IGF-Trap	Rapid2 stimulator (Magstim Ltd., Oxford, UK) Repetitive stimulation, modified butterfly TMS coil with a customizable tilt angle between wings rTMS: 130% of the resting motor threshold (rMT); 50 s train duration, 60 s intertrain interval, 5 trains, and 250 pulses in total per animal Frequency: 1 Hz Duration: 57 days with 2 sessions/week	Tumor size NSS Brain MRI scan	**Rats:** No effects in the NSS **IGF-Trap + rTMS** Partially inhibited tumor growth Reduced tumor size **Mice:** Transient disruption of the BBB rTMS increased the antitumor effect of IGF-Trap during the early phases of tumor growth

Caption: BBB: blood–brain barrier; IGF-Trap: IGF signaling inhibitor; IHC: immunohistochemistry assay; IL-2: interleukin-2; MRI: magnetic resonance imaging; NSS: neurological severity score; rMT: resting motor threshold; RT-qPCR: reverse-transcriptase polymerase chain reaction; s: seconds; T: Tesla; TNF-α: tumor necrosis factor-α; TMZ: temozolomide; TUNEL assay: terminal dUTP nick-end labeling.

## 3. Data Retrieved

### 3.1. Repetitive Magnetic Stimulation (rMS)

**Figure 1:**
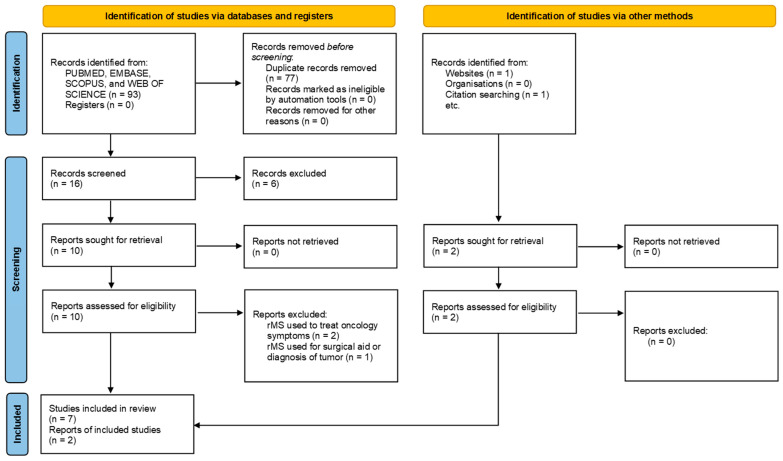
PRISMA flowchart depicting the search results for rMS.

A total of 93 studies were identified initially (Figure 1). Following the removal of duplicates, 16 studies were identified for screening. After the title and abstract review, 16 full-text articles were assessed for inclusion and exclusion criteria. Following full-text review and resolution of conflicts, nine articles (standard rMS = 8; patterned rMS = 1) met the inclusion criteria. The excluded studies presented rMS as a treatment for symptoms such as pain and major depression [11]. Some studies also highlighted how rMS can be used for imaging diagnosis and as an adjunct to surgery to remove tumor masses [45].

By assessing the nine selected studies, we found three in vitro and two conducted in animal models, while the remaining four include both cellular and animal-based analyses (Table 1). Figure 1 shows the PRISMA flow diagram displaying the search results and process.

#### 3.1.1. rMS Stimulation Devices and Protocols

Most of the studies used circular or elliptical coils (*n* = 7), which are characteristic of clinical research, and none of the studies presented a specific device for use in cells or rodent models, only customized devices. Two of the studies used the same stimulation device, the Magstim Rapid2 Plus (Magstim Ltd., UK) [46,51]. In addition, the authors used the same rTMS device and stimulation equipment, the customized rMS (Bicon-1000Pro, Mcube Technology, Seoul, Korea) [47,48]. Yamaguchi and colleagues used the same equipment, the magnetic stimulator (Nihon Kohden Co., Tokyo, Japan) [49,50].

Ashdown et al. [45] employed sequential 50 and 385 Hz oscillating magnetic fields to evaluate cell integrity. Furthermore, standard microscope slides were used to establish a platform for adjusting the elevation of the plated cells relative to the magnetic field.

The majority of the studies implemented a low-frequency protocol, revealing that it exerts an inhibitory effect. Lee et al. [48] substantiate this hypothesis, confirming the disparity between high and low frequency in a neuroblastoma cell line.

The duration of each stimulation session varied across the selected studies, with the shortest being 80 s [50] and the longest lasting 360 min [38]. Regarding the treatment period, Tatarov et al. [38] reported a 4-week stimulation period, with sessions occurring daily. Three trials employed a total treatment period of three days [47,48,50]. Only one study failed to disclose consecutive intervention days, although it performed stimulation for five days [51]. Yamaguchi et al. [49] also developed three distinct protocols concerning the duration of each stimulation session and the pulse pattern employed.

Heng et al. [46] was the only study among the eight selected publications that included five experimental protocols of intermittent rMS (iRMS) and two of burst rMS (bRMS) applied to cell monolayers in a single treatment session, distinguishing it from conventional rMS protocols.

#### 3.1.2. In Vitro rMS Studies [Table 1]

Of the nine selected studies, seven applied cellular analysis, indicating that a diverse array of tumor cell lines was employed [23,45,46,47,48,49,50]. Jo et al. [47] and Lee et al. employed the Neuro-2a (N2a) and the N1E-115 murine neuroblastoma cell lines [47,48], respectively. Jo et al. [23] analyzed the effects of rMS using three glioblastoma cell lines (U87MG, TS15-88, and TS21-117). Heng et al.’s [46] research included two cell lines: hepatocellular carcinoma (HuH7) and colorectal cancer (HCT116). The research conducted by Yamaguchi et al. [49,50] used the B16-BL6 murine melanoma cell line, examined both in vitro and in vivo for inoculation in mice. For analysis in mice, Tatarov [38] used the EpH4-MEKBcl2 metastatic mouse breast tumor cell line. In Ashdown’s [45] study, several cell lines were used in different experiments, including A549 (human lung cancer cells), Lewis lung carcinoma (LLC), human lung microenvironment cells, with human lung lymphatic microvascular endothelial cells (hLECs) being the only non-immortalized culture used, and MDA-MB-231 human breast carcinoma cells.

#### 3.1.3. In Vivo rMS Studies [Table 2]

All studies used animals inoculated with tumoral cells. Five of these studies employed only mice [23,38,47,49,50], whilst only Perrino et al. [51] included both mice and rats as the species under investigation.

#### 3.1.4. Outcome Assessments in rMS Studies

For cell viability analysis, the colorimetric 3(4,5-dimethylthiazol-2-yl)-2,5-diphenyl tetrazolium bromide (MTT) viability assay was used in three studies [41,44,45], and Jo et al. used the Cell Counting Kit 8 (CCK-8) test [23,47]. Ashdown et al. evaluated cell viability with a luminescence-based ATP production assay, which was assessed 4 h post-exposure. In this same investigation, cell proliferation was assessed by the number of cells, determined by the use of an automated hemocytometer and counted for 4 days [45].

Jo et al. [47] and Lee et al. [48] investigated cell proliferation through distinct methodologies; the former employed a colony formation assay, which is particularly suited for assessing cell survival based on the capacity of an individual cell to form a colony, while the latter employed microscopy to analyze diverse experimental groups. These studies conducted molecular analyses employing high- and low-frequency rMS, respectively. Lee et al. [48] reported the expression levels of the proteins brain-derived neurotrophic factor (BDNF), glial cell line-derived neurotrophic factor (GDNF), neurotrophin-3 (NT-3), and platelet-derived growth factor (PDGF), along with Akt and ERK protein expression levels via Western blotting. Jo et al. demonstrated protein expression using Western blotting, both in vitro and in vivo, focusing on the AKT, ERK, and Wnt/β-catenin signaling pathways and employing gene ontology analysis to study the biological processes downregulated by low-frequency rMS in neuroblastoma [47].

In addition, Yamaguchi et al. measured specific cytokines, IL-2 (interleukin 2) and TNF-α (tumor necrosis factor-α), in female C57BL/6J mice inoculated with B16-BL6 murine melanoma cell line to analyze the immunomodulatory effect induced by rTMS using the Biosource Immunoassay Kit (Biosource Co., Camarillo, CA) [50].

Five in vivo studies presented tumor size as an outcome assessment for tumor progression. Jo et al. [47] and Yamaguchi et al. [50] investigated the tumor mass by assessing tumor weight after euthanasia. Three of these studies presented an in vivo imaging system to evaluate the tumor mass. Yamaguchi et al. [49] used a computational model constructed from a set of magnetic resonance images. Tatarov et al. [38] implemented whole-body bioluminescence imaging to monitor the tumor growth. Perrino et al. [51] used a volumetric tool (Carestream Vue 12.1 Carestream Health, Rochester, NY, USA), measured by an experienced radiologist.

To detect signs of apoptosis, the TUNEL assay (terminal dUTP nick-end labeling) was performed in two studies [38,47]. Jo et al. [47] conducted the assay on Neuro-2a (N2a) cells as well as in vivo, using the DeadEnd™ Fluorometric TUNEL System (Promega, Madison, WI, USA). TUNEL-positive cells were increased in the low-frequency group compared to those in the sham group [47]. Tatarov et al. performed it exclusively in an in vivo model [38], showing by TUNEL assay that the stimulated animals presented fragmented cell nuclei, indicating apoptosis.

Ashdown et al. [45] analyzed the cell surface using a scanning electron microscope (Zeiss—Gemini FE-SEM), intending to complete the data assays of dead cell protease activity to analyze the cell membrane integrity. Additionally, the authors evaluated glycan enzymatic digestion. The experiment aimed to investigate how manipulation of specific molecules on the surface of cells, such as heparan sulfate (HS) and sialic acid, affects their interactions with proteins and overall cellular behavior. Heparin Lyase III (H’ase III) was used to digest HS on the cell surface, and fibroblast growth factor 2 (FGF-2) binding was measured to assess how HS removal affects protein binding. In addition, sialidase was applied to remove sialic acid to further modify the cell surface. By examining these mechanisms, the experiment explored how changes in cell surface molecules affect cellular responses such as growth, adhesion, and interaction with external signals.

To analyze the morphology of the tumor mass, histopathology was performed in two studies [38,50] using the bioluminescent assay. However, with the same objective, Jo et al. [42] presented the results by immunohistochemistry, with staining using an antibody against Ki-67.

Perrino et al. [51] was the only study that employed animal behavioral analysis, specifically the neurological severity score (NSS).

### 3.2. Static Magnetic Stimulation (sMS)

**Figure 2:**
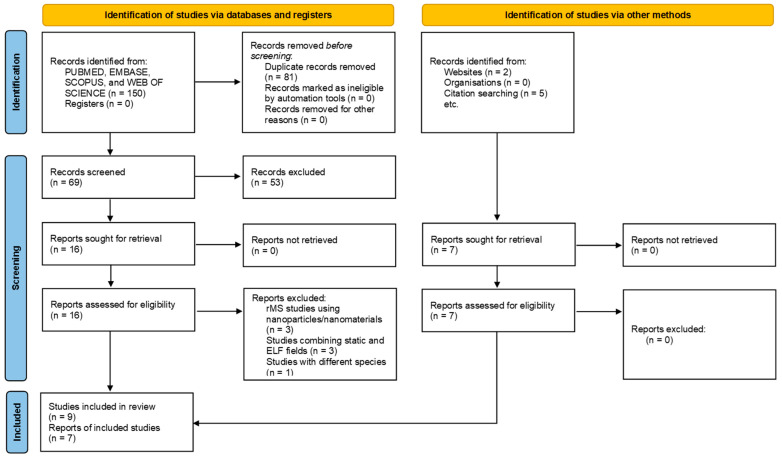
PRISMA flowchart depicting the sMS search results.

The search strategy used included one hundred and fifty papers (Figure 2). Following the exclusion of 81 papers, 69 papers were analyzed based on their title and abstracts. Three independent reviewers conducted the screening, and in cases where there were disagreements over classification, the relevant 16 studies from the study were reviewed. Studies were excluded mostly due to inappropriate interventions or incorrect populations, specifically when the studies were clinical reports. Studies employing sMS alongside extremely low-frequency stimulation as an intervention were not included in the review, as they involve two concurrent treatments, making it impossible to isolate the effects of a single stimulation. A total of sixteen studies were eligible and extracted for this scoping review. Figure 2 shows the PRISMA flow diagram displaying the search results and process.

#### 3.2.1. sMS Protocol and Stimulation Devices

Neodymium magnets have been employed in seven studies [25,26,52,53,54,55,56], establishing them as the most widely used tool. Zhang et al. [57] and Chen et al. [58] performed their studies with a solenoid made of copper wire wound around a ring with a radius of 4 cm and length of 54.7 cm. Jalali et al. [59] employed iron blades as the field conductor, while Zhao et al. [60] employed 16 T superconducting magnets for an identical purpose. In Kamalipooya et al., the exposure to sMS was performed using a locally designed sMF generator, consisting of two coils and a switching power supply [61]. Yang et al. selected two exposure systems to experiment with different intensities; the 150 mT system was composed of 10 small magnetic plates, each containing eight north polar magnets, whose strength was 500 mT and which formed the upward 150 mT magnetic plate on the surface of mice. The 22 T sMS was generated by a water-cooled magnet (WM2) in the Chinese High Magnetic Field Laboratory [62]. Sun et al. did not specify the static magnetic stimulation equipment [37].

The majority of studies employed an average magnetic field intensity of 0.2 T, which is considered moderate. Furthermore, Gray et al. [55] conducted their study at a moderate level of 0.11 T, which was lower than the intensity employed by the majority of other investigations included. On the other hand, Jalali et al. [59] employed much reduced intensities of 0.01 T, 0.015 T, and 0.025 T in their in vitro experimental methodology. Only Yang et al. used a high magnetic field intensity of 22 T [62].

The research applying sMS as the primary intervention exhibited variability in session duration, similar to the rMS studies. Medeiros et al. [25] reported a minimum session time of 60 min, while Zhao et al. [60] recorded a maximum session duration of 7 days. The remaining studies usually scheduled their sessions with an average interval of 48 h. Most research employed a Gaussmeter or Teslameter to quantify the intensity of the magnetic field, except for Chen et al. [58], Teodori et al. [54], and Gray et al. [55].

#### 3.2.2. In Vitro sMS Studies [Table 3]

The inclusion of studies indicated that 13 out of the selected ones utilized cell culture as their methodology. Medeiros et al. [25,26] employed the human neuroblastoma cell line SH-SY5Y as the principal model in both studies, with the initial paper using SH-SY5Y cells differentiated into dopaminergic neurons with retinoic acid [25]. Furthermore, this study also made use of a non-neuronal tumor line, the human vaginal malignant melanoma HMVII, and adipose-derived mesenchymal stem cells for evaluation of the sMS effect. Kim et al. [63] and Sun et al. [37] also used neural tumor cells, glioblastoma U87MG and U251MG lines, respectively. Teodori et al. [54] used glioblastoma primary cell culture from four patients. Chen et al. [48] and Zhang et al. [57] used the K562 cell line to analyze the sMS effects in myeloid leukemia. Tenuzzo et al. [53] analyzed the sMS effects in different cell lines, such as isolated human lymphocytes, thymocytes from mice, FTRL-5 (a cloned line of thyroid differentiated cells), U937 (monoblastic cells), G23DO cells (T hybridoma), HeLa (human cervical cancer), and HepG2 (human hepatocellular carcinoma). Chen et al. [52] also selected HepG2 to analyze the effects. Kamalipooya et al. [61] utilized the HeLa cell line and the Hu02 cell line to compare the sMS effects. Zhao et al. [60] employed two tumor cell lines from distinct animal species: K7M2 from murine osteosarcoma and MG63 from human osteosarcoma. Zafari et al. [27] and Jalali et al. [59] investigated the sMS effects in cells with pharmacological resistance, using the A2780-CP human ovarian cancer cell line, which is resistant to cisplatin, in comparison to the non-resistant A2780 cell line (Table 3).

#### 3.2.3. In Vivo sMS Studies [Table 4]

Four sMS studies utilized rodents. Gray et al. [55] used female B6C3F1 mice, approximately 5 weeks old, inoculated with uniform fragments of murine mammary cancer. In the same study, static electric stimulation was employed; however, only the group that received sMS as an intervention was evaluated. Additionally, Zhao et al. [60] used four-week-old male Balb/c mice inoculated with osteosarcoma stem cells (OSCs). Yang et al. [62] used a total of 24 25-day-old (~16 g) male SPF BALB/c (Nu/Nu) mice with the A549 cell line inoculated. Only one used Syrian golden hamsters bearing syngeneic A-Mel-3 melanomas to perform the experiment [52] (Table 4).

**Table 3:** Studies employing static magnetic stimulation (sMS) as a potential antitumoral therapy in in vitro models.

First Author (Year)	Cell Line	Stimulation Protocol	Outcome	Main Results
Medeiros et al. (2020) [25]	SH SY5Y (neuroblastoma cell) SH SY5Y differentiated cells HMV (human vaginal malignant melanoma cells) Mesenchymal cells	A custom-made stand for attaching 24-well standard plates was used for sMS stimulation NdFeB magnets Intensity: 0.1, 0.2, and 0.3 T Duration: 60 min and 24 h	Cell viability (MTT assay) Cell death (PI/Hoechst; Annexin-V/PI staining) Cell cycle analysis (PI staining) PCR *BDNF* gene expression	**sMS** Reduced viability in SH-SY5Y cells Increase in necrotic cells No effects on the cell cycle No variation in *BDNF* expression in SH-SY5Y cells subjected to SMS, except for the analyses conducted between 24 and 48 h
Medeiros et al. (2023) [26]	SH-SY5Y neuroblastoma cell HMV (human vaginal malignant melanoma cells) Mesenchymal cells	A custom-made stand for attaching 24-well standard plates was used for sMS stimulation NdFeB magnets Intensity: 0.3 T Duration: 6, 12, 24, 36, 72 h, and 6 days	Cell viability (MTT assay) Cell death (Annexin-V/PI staining) Cell cycle (DNA content) Proliferation (CFSE assay) Autophagy (acridine orange staining) Mitochondrial mass (MitoTracker Red)	**sMS** Reduced cell viability at 24 h and 6 days Decreased autophagy and cell proliferation after 6 days Reduced mitochondrial mass after 6 days No effect on cell cycle arrest or cell death
Zafari et al. (2024) [27]	Cisplatin-sensitive (A2780) and -resistant (A2780CP) ovarian carcinoma cells Groups (both cell types): ○ (1) Without treatment ○ (2) sMF ○ (3) Cisplatin ○ (4) sMF + cisplatin	sMF via a local generator (two wire coils with 3.0 mm and inductance of 2 H, resistance of 3 Ω, and a heat resistance of up to 200 °C) Intensity: 15 T Duration: 24, 48, and 96 h	Comet assay Cell cycle analysis Apoptosis assay Annexin V/PI PCR	**sMF + cisplatin** Increased DNA damage in both sensitive and resistant cell lines Increased the mortality of cells via necrosis and mostly apoptosis Increased the expression levels of apoptotic genes (*TP53* and *P21*) Minimal effect on the expression of *BCL2* Increased *CTR1* gene expression
Sun et al. (2025) [37]	U251 and U87 glioblastoma cells TGF-β1 (10 ng/mL) Groups (both cell line): ○ (1) Without treatment ○ (2) TGF-β1 ○ (3) sMF ○ (4) sMF + TGF-β1	sMF Intensity: 0.1 ± 0.01 T Duration: 72 h	Cell cloning and colony formation assay EdU proliferation assay Apoptosis assay Migration and invasion assays Western blot	**TGF-β1** Altered cell morphology Increased proliferation Promoted migration and invasion tumor cell Reduced apoptosis **sMF** Minimal impact on cell morphology Increased apoptotic cell proportion in both cell lines Decreased protein levels of mesenchymal markers N-cadherin and β-catenin **sMS + TGF-β1** Reduction in cell migration and invasion Increase in apoptosis Decreased protein expression of mesenchymal markers (N-cadherin and β-catenin) Reduced the expression of MMP-2
Chen et al. (2010) [58]	K562 human erythroleukemia cell line Groups: ○ Control ○ sMF alone ○ Cisplatin 5 mg/mL ○ Cisplatin 10 mg/mL ○ Cisplatin 20 mg/mL ○ sMF + cisplatin 5 mg/mL ○ sMF + cisplatin 10 mg/mL ○ sMF + cisplatin 20 mg/mL	sMF: solenoid made of copper wire wound around a ring Intensity: 8.8 mT Duration: 12 h	MTT assay: cell viability Cell cycle—PI by flow cytometry Comet assay FCM analysis Atomic force microscopic observation	**sMF alone** Increased cell death induced by cisplatin reducing the effective concentration from 20 mg/mL to 10 mg/mL **sMF + cisplatin** Inhibited cell proliferation Caused severe damage to cellular DNA (thinning, increased breaks and cross-links)
Chen et al. (2018) [52]	HepG2 (human hepatocellular carcinoma cell line) Groups (all with or without sMF): ○ Control ○ 0.5% DMSO ○ Capsaicin 25 µM ○ Capsaicin 50 µM ○ Capsaicin 75 µM	sMF: cylindrical permanent magnets derived from the rare-earth material neodymium iron boron Intensity: ~0.5 T Duration: 72 h	Cell viability—MTT assay Cell apoptosis—Anexin/PI by flow cytometry Western blot—Bax and Bcl-2 analysis Immunofluorescence microscopy Rhod-4 intracellular calcium assay	**sMF** Did not cause anticancer effect **sMF + capsaicin** Increased the anticancer effect Increased the binding efficiency of capsaicin for the TRPV1 channel Intensified capsaicin-induced mitochondria-dependent apoptosis pathway Increased the concentration of intracellular calcium
Kamalipooya et al. (2017) [61]	Human cervical cancer (HeLa) cell line and normal skin fibroblast cells (Hu02) Groups: ○ (1) Without treatment ○ (2) Cisplatin ○ (3) sMF ○ (4) Cisplatin + sMF	sMF: custom-designed generator, consisting of two coils and a DC switching power supply Intensity: 7, 10, and 15 mT Duration: 24 and 48 h	Cell viability—MTT assay Determination of intracellular ROS—DCF-DA Cellular ROS Estimation of lipid peroxidation—by measuring malonyldialdehyde (MDA)	**Cisplatin** Reduced the viability of cells with the increase in dose Increased the intracellular ROS production **sMF** Decreased cell viability with the intensities of 7, 10, and 15 mT (24 and 48 h) Higher membrane lipid peroxidation in cancer cells (HeLa) at both times but higher at 48 h **sMF + cisplatin** 10 mT for 48 h decreased the viability percent of HeLa cells by about 89% compared to untreated cells Increased ROS formation in both cell types Sensitized human cervical cancer cells to cisplatin through ROS accumulation
Jalali et al. (2019) [59]	A2780 and A2780-CP human ovarian cancer cells Group (both cell line): ○ (1) Without treatment ○ (2) sMF alone ○ (3) Cisplatin 1, 10, 50, 100, and 500 µM ○ (4) sMF + cisplatin	sMF: locally designed generator Intensity: 10, 15, or 25 mT Duration: 24, 48, or 96 h	Cell viability (MTT assay) IC50 (cisplatin sensitivity) Cisplatin uptake (ICP-OES)	**sMF + cisplatin** Did not affect cell viability No significant increase in cisplatin sensitivity in both cell lines **Cisplatin alone** Decreased viability in sensitive and resistant cells (more pronounced in sensitive) A2780 cells were more cisplatin-sensitive Increased cisplatin uptake
Kim et al. (2016) [63]	Human glioblastoma U87MG and U251MG cells	sMF: Hirst Magnetic Instruments Ltd., Falmouth, England Intensity: 1.4 to 2.6 T Duration: 48 ± 4 h	WST-1 cell viability assay Apoptotic signal assessment of viability—Annexin V test Immunocytochemistry Protein expression related to the cell cycle (cyclin B1 and Cdk1)	**sMF** Decreased cell viability Reduced Cdk1 protein expression, affecting cell cycle regulation No effects on apoptosis
Tenuzzo et al. (2006) [53]	U937 HeLa HepG2 (human hepatocellular carcinoma cells FRTL-5 cells	sMF: neodymium magnetic disks (Calamit Ltd., Milano, Italy) Intensity: 6 mT Duration: 24 and 48 h Apoptosis-inducing agents: cycloheximide 10^2^ M, H_2_O_2_ 10^3^ M, puromycin 10^6^ M, heat shock 43 °C	Cell viability—MTT assay Cell growth rate Apoptosis assay—Hoechst-33342/propidium iodide (PI) or hematoxylin/eosin-stained cells Scanning electron microscopy Measurements of Ca^2+^ levels	**sMF** Promoted apoptosis and mitosis but not necrosis or modifications of the cell shape Recovered from apoptosis induced by pro-apoptotic drugs Increased intracellular Ca^2+^ levels
Teodori et al. (2002) [54]	Primary culture of human glioblastoma Apoptosis induced by etoposide (VP16) 100 M or heat shock (HS) of 42.5 °C for 24 h	sMF: metal magnetic disks Intensity: 6 mT	Microfluorimetry: intracellular Ca^2+^ concentration Apoptosis assay: Hoechst 33342 and propidium iodide (PI) and Annexin V/fluorescein (FITC)	**sMF** Increased [Ca^2+^] influx Non-apoptogenic activity Reduced the extent of stress-induced apoptosis Increased cell survival Reduced apoptosis, cell line-dependent
Zhang et al. (2014) [57]	K562 human erythroleukemia cell line Groups: ○ (1) Control ○ (2) sMF ○ (3) Cisplatin 10 mg/mL ○ (4) sMF + cisplatin	sMF: solenoid made of copper wire wound around a ring Intensity: 8.8 mT Duration: 4, 8 or 12 h	Cell viability: MTT assay HPLC analysis FAAS analysis Analysis of P-glycoprotein expression using flow cytometry	**sMF** No effects **Cisplatin 10 mg/mL** Induced the expression of P-gp in K562 cells **sMF + cisplatin** Significantly inhibited the metabolic activity of K562 cells, while DDP or sMF alone did not Increased the concentration of intracellular DDP Reduced the ratio of K562 cells with P-gp expression
Zhao et al. (2021) [60]	Murine osteosarcoma cell line K7M2 and human osteosarcoma cell line MG63	sMF: 16 T superconducting magnet 400–500 mm away from the center with a spatial gradient of 2.09 T/m in the radial direction Intensity: 0.2–0.4 T	Light microscopy Crystal violet staining Cell Counting Kit 8 (CCK-8) assay ROS detection H&E staining IHC IF Western blot siRNA transfection TEM Fe^2+^ detection assay	**sMF** **After 24 h** Increased ROS levels **After 7 days** Promoted the release of ferrous iron (Fe^2+^) Induced proliferation and tumor sphere Elevated the expression of ferritinophagy-related markers *LC3-II* and *NCOA4* receptor and autophagosomes of K7M2 and MG63 Downregulated the expression of *FTH1* and enhanced the levels of *NCOA4*

Caption: BDNF: brain-derived neurotrophic factor; FAAS: flame atomic absorption spectrophotometer; Fe^2+^: ferrous iron; H&E: hematoxylin−eosin; HPLC: high-performance liquid chromatography; IF: immunofluorescence; IHC: immunohistochemistry/immunohistochemistry assay; MMP-2: matrix metalloproteinase 2; MTT: 3-(4,5-dimethylthiazol-2-yl)-2,5-diphenyl-2H-tetrazolium bromide; ROS: reactive oxygen species; siRNA: small interfering RNA transfection; sMS: static magnetic stimulation; sMF: static magnetic field; T: Tesla; TEM: transmission electron microscopy; TGF-β1: transforming growth factor β1.

**Table 4:** Studies employing static magnetic stimulation (sMS) as a potential antitumoral therapy in in vivo models.

First Author (Year)	Animal Model	Stimulation Protocol	Outcome	Main Results
Gray et al. (2000) [55]	Female B6C3F1 mice transplanted with 16/C mammary adenocarcinoma cells Groups: ○ (A) Adriamycin 10 mg/kg ○ (B) Adriamycin + SEAC ○ (C) Adriamycin + NSEF ○ (D) Adriamycin + sMF	**sMF** Neodymium permanent magnets, 12.7 mm diameter by 5.1 mm long, with a 1.08 T flux density (The Magnet Source Inc., Miami, FL, USA) Intensity: 110 mT Duration: one 4 h session **SEAC and NSEF** 15,000 V DC supplies (Spellman High Voltage Electronics Corp., Plainview, NY, USA). Positive potential (#MP15P) and negative potential (#MP15N) Output ripple of less than 10 mV peak to peak Duration: one 4 h session	Tumor size on days 7, 11, 14, 17, and 20 Odds ratio vs. group A	**Adriamycin** No effects on tumor regression **SEAC + Adriamycin** Increased the tumor regression vs. Adriamycin only (7:1, up to D14) **NSEF + Adriamycin** Increased the tumor regression vs. Adriamycin only (10:1, up to D17) **sMF + Adriamycin** Increased the tumor regression vs. Adriamycin only (59:1, up to D20)
Strelczyk et al. (2009) [56]	Syrian golden hamsters bearing syngeneic A-Mel-3 melanomas	sMF was generated by a cylindrical permanent magnet derived from the rare-earth material neodymium iron boron (NdFeB; 250/175 h) Magnet Fabrik Schramberg, Schramberg, Germany Intensity: 586 mT Duration: 3 h	Tumor angiogenesis (in vivo fluorescence microscopy for functional vascular density, red blood cell velocity, and vessel diameters) Tumor growth Histology (H&E staining) Animal behavior and body weight	Prolonged exposure to sMS retarded the growth of solid tumors in vivo sMF impaired tumor angiogenesis Reduced intratumoral functional vascular density and red blood cell velocity Increased edema and impaired intercellular adhesion No effects on the behavior or body weight
Yang et al. (2023) [62]	Male SPF BALB/c (Nu/Nu) mice inoculated with A549 cell line (human lung adenocarcinoma cells) All groups received platycodin D 2 mg/kg (PD) Groups: ○ (1) Sham ○ (2) Control ○ (3) sMF 22 T ○ (4) sMF 150 mT	sMF: water-cooled magnet (WM2) in Chinese High Magnetic Field Laboratory (Hefei, China) Intensities: moderate and ultra-high: 150 mT and 22 T Duration: 6 days	Elevated plus-maze test Open field test Non-invasive pulse oximetry test Complete blood count and blood biochemistry test H&E staining: Heart, liver, spleen, lung, kidney, and tumor tissues RNA extraction and RNA-sequencing (RNA-seq)	**sMF** No differences in body weight, water intake or food consumption among PD and sMF groups No effect in routine blood, blood biochemistry test, H&E staining or organ index PD ameliorated dysphoria in mice, but sMFs reduced this effect The antitumor effect of 22 T was 3.6 times higher than that of PD The sMF antitumor effect of 150 mT was only 1.56-fold greater than that of PD RNA-seq showed sMFs and PD synergistically targeted genes associated with tumor growth, inflammation and neurological diseases
Zhao et al. (2021) [60]	Osteosarcoma stem cells (K7M2 OSCs) into tumor-bearing BALB/c male mice	sMF using a 16 T superconducting 400–500 mm away from the center with a spatial gradient of 2.09 T/m in the radial direction Curative treatment: sMF after tumor development in vivo Preventive treatment: injection of cells after sMF exposure in vitro Intensity: 0.2–0.4 T	ROS detection H&E staining IHC IF Western blot siRNA transfection TEM Fe^2+^ detection assay	Did not affect the tumor volume or mass (curative and preventive treatment) Increased lung metastasis (only preventive treatment)—higher levels of Ki-67

Caption: Fe^2+^: ferrous iron; H&E: hematoxylin−eosin; IF: immunofluorescence; IHC: immunohistochemistry/immunohistochemistry assay; NSEF: negative static electric field; SEAC: static electric alternating current; ROS: reactive oxygen species; siRNA: small interfering RNA transfection; sMS: static magnetic stimulation; sMF: static magnetic field; T: Tesla.

#### 3.2.4. Outcome Assessments in sMS Studies

The most frequently observed test in the selected articles was related to cell viability, with the MTT assay identified in eight articles [25,26,52,53,54,57,58,59,61,62]. To validate the hypothesis of magnetic stimulation as a possible antineoplastic treatment, the second most frequently performed test was the assessment of cell death by apoptosis assay by Hoechst 33342 or/and propidium iodide (PI) and Annexin V/fluorescein (FITC) [25,26,52,63]

In vivo investigations reveal a consistent pattern of assessments, including Hematoxylin and Eosin (H&E) staining of tumor tissues, tumor growth, animal behavior, and body weight [6,7,8,9,10,11,12,13,14,15,16,17,18,19,20,21,22,23,24,25,26,27,28,29,30,31,32,33,34,35,36,37,38,39,40,41,42,43,44,45,46,47,48,49,50,51,52,53,54,55,56,57,58,59,60,61,62,63]. Yang et al. [63] conducted more comprehensive testing pertaining to biochemistry and RNA sequencing (RNA-seq).

To evaluate the potential impact of sMS and cellular mechanisms, intracellular Ca^2+^ concentration [52,53,54] was employed to elucidate cell signaling and modulation of apoptosis; production of reactive oxygen species (ROS) such as DCFH-DA (dichlorodihydrofluorescein diacetate) and membrane lipid peroxidation, related to lipid membrane damage [61]; mitochondrial mass (MitoTracker Red)related to late cell autophagy as a mechanism of cell survival [26]. Furthermore, electron microscopy (TEM and SEM) was employed for detailed observation of morphological and ultrastructural changes at the subcellular level, including cell shape and membrane surface [53,58,61]. As molecular mechanisms were evaluated, Western blot analysis was performed for epithelial–mesenchymal markers such as N-cadherin, β-catenin, and matrix metalloproteinase-2 (MMP-2), as well as Bax and Bcl-2 [37,52,63].

#### 3.2.5. MS Combined with or Compared with Antitumoral Drugs

Ten studies reported another type of antitumoral treatment in combination with MS [27,37,51,52,53,54,55,56,57,58,59,61,62]. Five of these studies used cisplatin as the primary antineoplastic treatment, in varying concentrations, ranging from 25 µM to 2 mg/kg [27,57,58,59,61]. Yang et al. [62] used platycodin D at a concentration of 2 mg/kg. Gray et al. [55] and Chen et al. [52] used Adriamycin (10 mg/kg) and capsaicin (25, 50, and 75 µM) to block TRPV1 channels, respectively. Tenuso et al. used cycloheximide (CHX; 10^−2^ M), H_2_O_2_ (10^−3^ M), puromycin (PMC; 10^−6^ M), and heat shock (HS; 43 °C) [53]. Teodori et al. used etoposide (VP16) or HS as apoptosis inducers [54]. Meanwhile, Sun et al. associated MS with the transforming growth factor β1 (TGF-β1), which is a key factor promoting proliferation, migration, and epithelial–mesenchymal transition (EMT) in glioblastoma (GBM) [37]. EMT may play a crucial role in tumor invasion and drug resistance. In addition, Perrino et al. [51] associated rTMS with IGF-Trap.

One study compared the effects of low-frequency antitumoral potential of rTMS with the oral chemotherapic temozolomide [23], assessing the expression of *FLNA*, *FLNC*, *Ki-67*, *EGF*, *EphA2*, *MMP2*, and *MMP9*, and the ERK/JNK/p38 and PI3K/AKT/mTOR pathways.

#### 3.2.6. Risk of Bias

Two investigators conducted the risk of bias analysis independently. Overall, there was high inter-rater agreement in the risk of bias assessment with both tools employed. Discrepancies were resolved through discussion and consensus. Using the QUIN risk of bias tool for in vitro studies [43], a 93% overall agreement was achieved across categories. Using the SYRCLE risk of bias tool for animal studies [44], a 97% overall agreement was achieved across categories.

The risk of bias assessment indicated a mixed overall methodological quality across the included in vitro and in vivo studies.

**Figure 3:**
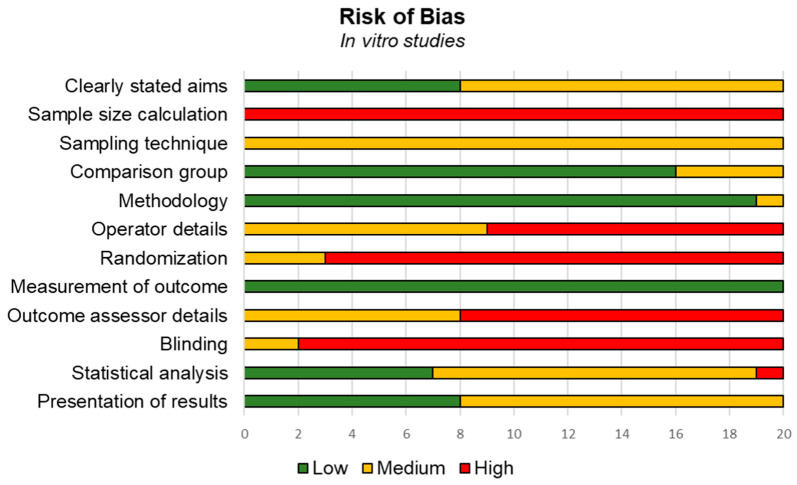
Risk of bias results using the QUIN tool [43].

In in vitro investigations, several studies demonstrated medium to high risk of bias. The final score of the included studies was 49.79%, meaning that they presented a high risk of bias overall according to the QUIN tool (44). High risk of bias was observed for “sample size calculation”, “operator details”, “randomization”, “outcome assessor details”, and “blinding”. Medium risk of bias was observed for “clearly stated aims/objectives”, “detailed explanation of sample technique”, “statistical analysis” and “presentation of results”. All other criteria had low risk of bias, named “details of comparison group”, “detailed explanation of methodology” and “method of measurement outcome” (Figure 3).

**Figure 4:**
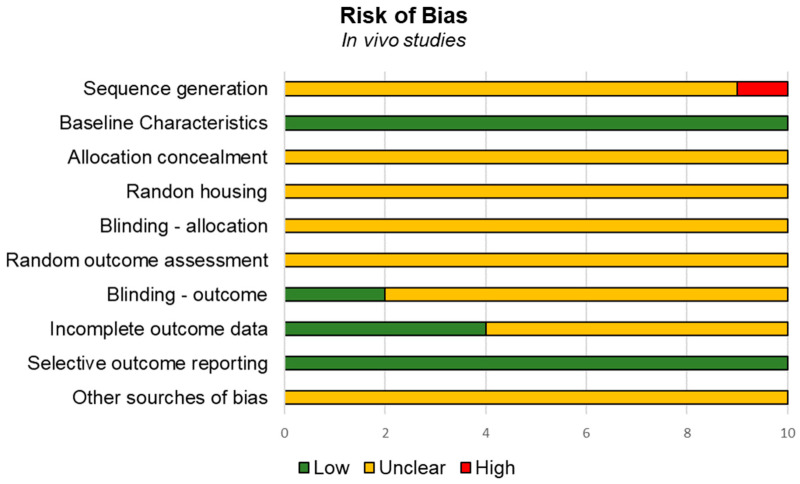
Risk of bias results using the SYRCLE tool [44].

In in vivo investigations, several studies demonstrated medium to high risk of bias according to the SYRCLE tool [44]. High risk of bias was observed for the domain “sequence generation”. Unclear risk bias was detected for the domains “allocation concealment”, “random housing”, “blinding-allocation”, “random outcome assessment”, “blinding-outcome assessment”, “incomplete outcome data”, and “other sources of bias”. Low risk of bias was observed for the domains “baseline characteristics”, and “selective outcome reporting” (Figure 4).

Overall, although a proportion of the studies were judged to be at low risk of bias, the presence of methodological limitations in key domains suggests that the findings should be interpreted with some caution.

## 4. Discussion

### 4.1. General Mechanisms and Therapeutic Potential

Neuromodulatory techniques have been examined for their efficacy in treating brain tumors, focusing on the ability to modulate certain neuronal activities within the central nervous system. Although numerous studies indicate a favorable safety profile for exposure to magnetic fields, the need for systematic long-term toxicological investigations in organs, including bone marrow and the reproductive system, remains emphasized.

The current scoping review classifies the majority of studies into two categories: static and repetitive MS, with the latter further divided into high- and low-frequency exposure. In this context, tumor proliferation is the most frequently mentioned outcome, and tumor suppression is the second most cited. In addition, the findings indicate that the biological responses to MS are significantly affected by tumor type and various stimulation parameters, including frequency, temporal pattern, and magnetic field intensity. Consequently, the characteristics of different cancer models require the development of stimulation methods aligned with tumor-specific features. Non-invasive magnetic techniques have shown promise in altering essential tumor characteristics, particularly membrane integrity, cellular viability, proliferation, and overall tumor growth, through the application of customized electromagnetic waves. The results suggest that some MS patterns may selectively target cancer cells, indicating it as an adjuvant therapy to the detrimental consequences associated with standard antitumoral treatment, likely facilitating the delivery of chemotherapy drugs across tumor cell membranes.

MFs induce biological effects that are significantly dependent on cell type and cellular conditions, intratumoral heterogeneity, and growth patterns [25,26,61]. A study using rMS demonstrated significant variability in cancer cells, validating that low-frequency protocols produce divergent effects across various tumor types; for instance, they reduce the viability of colorectal cancer cells while increasing it in hepatocellular carcinoma cells [46]. The results of sMS studies offer inconsistent outcomes: some indicate significant reductions in cell viability [63], whereas others demonstrate minimal or absent anticancer effects when applied in isolation [52,57,59]. The discrepancies in results suggest a considerable reliance on stimulation parameters, cell type, and experimental conditions, indicating that sMS is less robust as an independent method of treatment compared to rMS.

It is important to note that the included studies concerning this review address both in vitro and in vivo models that exhibit unique advantages and disadvantages. In vitro systems are cost-effective and highly controlled, facilitating tumor cell analysis without systemic influences; nonetheless, they poorly replicate the complexity of the human cellular environment, limiting their translational relevance. Conversely, in vivo investigations more accurately represent physiological conditions and enable the exploration of complex, indirect anticancer mechanisms; yet, they are more expensive, challenging to control experimentally, and may be limited by genetic and physiological differences between animal models and humans. The contradictory results between in vitro and in vivo studies found in this review emphasize the importance of animal models in understanding the therapeutic effects of MS on cancer and the necessity for prudence when generalizing data from cell culture to animal models.

### 4.2. Effects of Low-Frequency rMS

Accumulating evidence indicates that low-frequency magnetic stimulation exerts significant anticancer effects by modulating critical signaling pathways associated with cancer cell proliferation, survival, and tumor invasion [23,34,38,51,58,63]. In several experimental models employing neuroblastoma, glioblastoma, and colorectal cancer, low-frequency MS consistently decreases tumor growth, inhibits cell proliferation, and promotes apoptosis, with specific research indicating efficacy comparable to conventional chemotherapeutic drugs, such as temozolomide [23].

Mechanistically, these effects are mediated, in part, by the negative regulation of signaling pathways such as Wnt/β-catenin [47], which is frequently dysregulated in cancer and contributes to tumor growth, survival, and therapeutic resistance [64]. The suppression of Wnt/β-catenin is associated with an increase in apoptotic cell death and reductions in proliferative activity (reduced Ki-67 expression). Simultaneously, low-frequency rMS reduces the activation of receptor tyrosine kinases, including the cellular signaling factors *EGFR* and *EphA2* associated with tumor grade and metastatic cases, while also attenuating downstream PI3K/AKT/mTOR and *MAPK* (ERK/JNK/p38) signaling pathways. Consistent decreases in *Akt* and *ERK* expression [48] further support the involvement of these pathways in mediating the antiproliferative effects of magnetic stimulation.

Recently, Jo et al. [23] reported in an in vivo glioblastoma model that low-frequency rMS (0.5 Hz) also affects tumor cell invasiveness by modulating cytoskeletal organization and extracellular matrix remodeling. The downregulation of the actin-associated scaffold proteins *FLNA* and *FLNC* correlates with impaired cell migration and adhesion, whereas the lower expression of matrix metalloproteinases *MMP2* and *MMP9* is associated with decreased invasive capacity [65]. These molecular alterations provide a mechanistic foundation for the observed reductions in tumor invasion.

Interestingly, Heng et al. [46] demonstrated that pulsed low-frequency irMS reduces cell viability in HCT116 human colorectal adenocarcinoma (CRC), whereas high-frequency irMS and brMS increase it. In HuH7-hepatocellular carcinoma (HCC), neither rMS pattern affected viability. CRC and HCC cells exhibited opposing responses to the parameters of rMS, frequency, temporal patterns, and magnetic field flux density, indicating tumor type- and stimulation protocol-specific effects. The sensitivity of CRC cells to rMS (suppression of their viability by low-frequency iRMS) may depend on the effects of MF on molecular structures related to the cell membrane and, to a lesser degree, on the electron transport chain within mitochondria. On the other hand, in HCC cells, it seems likely that the effects of rMS on the mitochondrial electron transport chain may contribute to the reduction in viability. In addition, a study demonstrated that prolonged exposure to MF in a breast cancer mouse model resulted in tumor necrosis and significant growth inhibition, while not inducing histopathological damage to healthy tissues, suggesting low systemic toxicity and selective anticancer actions [38]. These studies suggest the need to optimize protocols and/or combine them with other therapies to improve outcomes dependent on tumor type.

In summary, low-frequency rMS demonstrates anticancer efficacy by simultaneously inhibiting carcinogenic signaling pathways, suppressing cytoskeletal and invasive mechanisms, and inducing apoptosis. These findings indicate the effectiveness of low-frequency rMS as a non-invasive therapeutic strategy for cancer and provide a potential molecular basis for subsequent preclinical and translational research.

### 4.3. Effects of High-Frequency rMS

High-frequency rMS appears to influence tumor biology through specific and context-dependent molecular mechanisms. High-frequency stimulation (10 Hz) markedly elevates the protein expression levels of *BDNF*, *GDNF*, *NT-3*, and *PDGF* in immortalized mouse neuroblastoma cells, alongside elevated *ERK* and *Akt* expression. In contrast, low-frequency stimulation (0.5 Hz) reduces *ERK* and *Akt* levels compared to control conditions [48], suggesting that the assessed parameters are frequency-dependent in their response to rMS.

Neurotrophic signaling pathways, which physiologically regulate neuronal survival, differentiation, and plasticity, are frequently co-opted by tumors to promote proliferation, angiogenesis, metastasis, and resistance to therapy. The aberrant activation of the ERK and Akt signaling pathways [48], triggered by growth and neurotrophic factors, suggests aggressive phenotypes, supporting sustained growth and adaptive capacity [66,67,68]. Furthermore, the simultaneous increase in neurotrophins, *PDGF*, and *ERK*/*Akt* signaling observed after high-frequency stimulation suggests a synchronized activation of pro-tumorigenic signaling networks rather than independent molecular alterations [48]. However, it is important to emphasize that results from Western blot analysis reflect protein expression levels and do not directly indicate functional activity or pathway flux, underscoring the need for complementary functional assays to confirm signaling activation.

Although direct effects have been observed in vitro, studies in vivo indicate that high-frequency MS may activate indirect anticancer pathways. Yamaguchi et al. [50] demonstrated that high-frequency rMS inhibited tumor growth and triggered significant tumor necrosis in mice injected with B16-BL6 melanoma cells, alongside elevated splenic levels of TNF-α; this pro-inflammatory cytokine is known for its antitumoral effects [69]. These data suggest that rMS may influence systemic or local immune responses, creating a tumor microenvironment detrimental to tumor survival. Tumor necrosis in this context can result from changes in perfusion, oxidative stress, inflammatory signaling, or immune-mediated cytotoxicity, processes that cannot be replicated in isolated cell culture systems [45]. Consistent with this interpretation, Yamaguchi et al. [49,50] found no significant effects of high-frequency rMS on B16-BL6 cell viability in vitro, emphasizing the limitations of reductionist models that exclude vascular, immune, and stromal components. These inconsistencies demonstrate that tissue context and systemic interactions significantly influence the biological effects of MS.

A study investigating the effects of MS on the membrane level provides further mechanistic insight. Ashdown et al. [45] demonstrated that brief exposure to pulsed and oscillating high- and very-high-frequency MS triggers the release of intracellular protease, leading to disruption of the membrane and reducing the survival of A549 human lung cancer cells. These cells have a highly charged, glycan-rich glycocalyx, while normal lymphatic endothelial cells (hLECs) are not affected by rMS. Similar effects were observed in breast cancer cells with sialic acid-rich glycocalyces, indicating tumor-specific susceptibility [45]. The findings show that short exposure to high- and very-high-frequency pulsed MS can directly compromise the membrane integrity in tumor cells in a glycocalyx-dependent manner, offering a potential non-signaling-based anticancer mechanism.

Current evidence indicates that MS yields intricate and frequency-dependent biological effects, including both direct modulation of tumor cell signaling as well as indirect pathways facilitated by immune activation and changes in the tumoral microenvironment. High-frequency stimulation can increase pro-survival signaling in isolated tumor cells through neurotrophic and growth factor pathways while simultaneously exerting anticancer effects in vivo through immune-mediated, vascular, or membrane-targeted mechanisms. This duality emphasizes the significance of stimulation parameters, tumor type, and experimental conditions in validating the overall biological effect of MS.

### 4.4. Effects of sMS/sMF

sMS exerts direct and varied effects on tumor cells, which are significantly affected by tumor type and exposure conditions. In glioblastoma U251 and U87 cell lines, the application of sMF inhibited cell migration and invasion, increased apoptosis, and downregulated proteins involved in cell adhesion and invasiveness, including N-cadherin, β-catenin, and MMP-2 [37]. Conversely, moderate (0.2–0.4 T) sMF exposure in osteosarcoma stem cell lines (K7M2 and MG63) for at least seven days promoted cell proliferation, increased tumorsphere formation, elevated Fe^2+^ content, and activated ferritin autophagy, suggesting ferritinophagy as a potential molecular target in cancer treatment [60]. These findings indicate that sMF can either suppress or stimulate tumor cell activity, highlighting the intricate nature of its biological impacts.

In vivo studies demonstrate that sMF can induce different effects on tumor growth. In tumor-bearing mice, exposure to preventive (sMF-pretreated K7M2 osteosarcoma stem cells) and therapeutic treatment by sMF did not change tumor volume or mass. On the other hand, mice that were injected with sMF-pretreated K7M2 osteosarcoma stem cells had an increased incidence of pulmonary metastasis, indicated by higher levels of Ki-67, whereas the sMF therapeutic intervention did not influence this outcome, suggesting that it may enhance pro-metastatic traits under certain conditions [60]. Still, another in vivo study (in Syrian golden hamsters) showed that tumors exposed to sMS decreased in size compared to untreated ones, likely resulting from tumor angiogenesis inhibition [56]. Overall, these findings reinforce that the effects of sMS depend on the context and highlight the importance of rigorously selecting the condition when evaluating sMF for tumor-related applications.

Two previous studies of our research group demonstrated that sMS reduces the viability of SH-SY5Y neuroblastoma cells after 24 h [25,26] and 6 days [26] of treatment, irrespective of conventional apoptotic mechanisms. Medeiros et al. [25] showed decreased cell viability, modified apoptotic, double-positive cell populations, and elevated necrotic cell counts, indicating that sMS may induce cellular stress that compromises the membrane integrity and homeostasis instead of facilitating regulated apoptosis. Medeiros et al. [26] corroborated this data by demonstrating reduced cell survival in SH-SY5Y cells following 24 h and 6 days of sMS therapy, with no changes in cell death markers or the cell cycle. sMS may impair cellular viability through sublethal pathways linked to mitochondrial changes, without triggering apoptosis or cell cycle arrest. Prolonged exposure decreases autophagy and cellular proliferation, significantly reducing the cellular adaptive capacity. Although the causal relationships cannot be established, the sMS’s effects on mitochondrial mass, autophagy, and proliferation indicate that it impairs cellular maintenance over time. Furthermore, the absence of viability changes in HMV-II cells suggests a cell-type-specific sMS response. This could be explained by distinct metabolic demands or stress-response mechanisms exhibited by neuronal and non-neuronal tumor cells. In summary, the studies by Medeiros et al. [25,26] demonstrate that sMS treatment diminishes SH-SY5Y cell viability via mitochondrial changes and cellular adaptation mechanisms, rather than by triggering apoptosis. In accordance with the hypothesis that apoptosis is not a major factor in cell viability reductions, Kim et al. [58] showed that the reduced viability in U87 and U251 glioblastoma cells following exposure to sMF was associated with decreased expression of cyclin-dependent kinase 1, rather than apoptotic signaling pathways.

### 4.5. Effects of MS Associated with Antitumoral Drugs

Numerous studies have demonstrated that sMFs may increase anticancer responses when combined with chemotherapeutic drugs [27,37,52,53,54,55,57,58,59,61,62,63]. More specifically, they have shown an increase in the efficacy of antitumoral drugs, such as cisplatin [47,48,57,59] and platycodin D [62]. Observed outcomes include increased antitumoral activity, reduced dose required [58], and reduced drug resistance in cancer cell lines [27]. Despite consistent results, the underlying mechanisms remain only partially comprehended and appear to include alterations in cellular drug resistance and tumor-associated signaling pathways.

Zhang et al. [57] demonstrate that sMF (8.8 mT) makes cisplatin more effective at killing K562 human erythroleukemia cells by modulating P-glycoprotein (P-gp)-associated drug resistance. While the isolated treatments did not affect cell viability, the combination of cisplatin and sMF resulted in marked inhibition of cell survival, indicating a stronger antitumoral effect. Furthermore, they observed a decrease in extracellular cisplatin concentrations, indicating increased intracellular drug accumulation. Cisplatin treatment alone has been shown to promote P-gp expression, suggesting an adaptive drug efflux response. Conversely, simultaneous treatment with sMF markedly decreases this induction, resulting in reduced *P-gp* expression relative to cisplatin alone. These findings demonstrate that sMF impairs cisplatin-induced efflux mechanisms, thereby increasing intracellular drug retention and its cytotoxic efficacy. A further effect of MS is a transient disruption of the blood–brain barrier, as noted by Perrino et al. [51]. Using rTMS, they observed an antitumoral effect by improving the delivery of anticancer molecules (IGF-Trap) to the brain in rodents injected with glioma cells intracerebrally, although tumors can progress or develop resistance to treatment over time.

A synergistic effect of MS and anticancer drugs was also observed by Yang et al. [62], providing molecular and translational evidence of the association of sMFs and PD in lung cancer models. Ultra-high sMFs (22 T) significantly improved the anticancer efficacy of PD compared to its administration alone, while moderate sMFs (150 mT) provided a more modest effect. Moreover, the combination of sMS with PD increased the inhibition of tumor-associated gene expression. A transcriptomic analysis indicated coordinated control of genes linked to tumor growth, inflammation, and neurological processes, revealing a complex mechanism of action. Furthermore, systemic toxicity was not significant as evidenced by behavioral, hematological, biochemical, and histological parameters, thus validating the safety of sMFs. These findings collectively reinforce the rationale for sMFs as a safe and effective adjunct in lung cancer treatment.

Corroborating the sMFs as an adjuvant approach to improving the antitumoral response in vivo, Gray et al. [55] showed that the exposure to static electric or sMFs increases the antitumor efficacy of Adriamycin in a murine mammary adenocarcinoma model. All field-exposed groups (both electric and magnetic) exhibited greater tumor regression compared with Adriamycin treatment alone [55]. In addition, Chen et al. [52] showed a synergistic effect of sMF with capsaicin, inhibiting HepG2 cancer cell growth through a mitochondria-dependent apoptosis pathway. Part of the mechanism by which sMF affects the capsaicin activity may involve a conformational change in the TRPV1 ion channel, likely making it more available to capsaicin and thereby changing the activation kinetics and the downstream apoptotic pathways, thus enhancing its anticancer efficacy.

Although some studies did not show significant cell death, sMS modulates cellular processes, including reductions in mitochondrial mass and autophagy [26] and increased ROS accumulation, lipid peroxidation [61], and intracellular calcium levels [53,54], all contributing to apoptotic signaling. A combined treatment utilizing static sMS (10 mT) and cisplatin demonstrated that the sMS exerted a more pronounced effect on HeLa cells compared to fibroblasts, selectively enhancing the sensitivity of human cervical cancer cells to cisplatin via increased accumulation of reactive oxygen species, primarily within the tumor cells [61]. In an in vitro study, the combination of sMF with cisplatin increased the expression of pro-apoptotic genes and promoted apoptosis and necrosis in both cisplatin-sensitive and cisplatin-resistant ovarian cancer cells [27]. sMF significantly suppresses TGF-β1-induced epithelial–mesenchymal transition (EMT) in glioma cells, diminishing migration and invasion [37]. Reduced expression of critical EMT markers was also observed, including N-cadherin, β-catenin, and MMP-2. In contrast to studies suggesting that sMF primarily affects cell viability through non-apoptotic mechanisms [25,26,63], evidence also indicates that sMF can increase apoptosis, suggesting disruption of EMT-associated survival pathways [37]. These data suggest that sMF can concurrently inhibit invasiveness and induce apoptosis, underscoring its potential as a therapeutic approach for inhibiting glioma growth.

Corroborating the low-frequency antitumoral potential of rTMS, Jo et al. [23] showed that it has a similar efficacy in decreasing cell viability and survival and increasing apoptosis in glioblastoma as the oral chemotherapeutic temozolomide. The observed effects were related to downregulation of the expression of *FLNA*, *FLNC*, and *Ki-67*, suppressing cell proliferation, and inhibition of the ERK/JNK/p38 and PI3K/AKT/mTOR pathways via downregulation of the *EGF* receptor and *EphA2* activation, inducing apoptosis. In addition, there was a reduction in *MMP2* and *MMP9* expression, which are highly overexpressed in tumors, contributing to the remodeling of the cellular cytoskeleton in the invasion and migration of glioblastoma.

### 4.6. Limitations to the Interpretation of Results

It remains challenging to consolidate and evaluate the findings of several studies examining the cellular and molecular effects of static and repetitive MS in cancer models due to the significant methodological variations employed in these investigations. Moreover, variations in cell and tumor models and experimental conditions employed both in vitro and in vivo were evident, with procedures differing significantly regarding field intensity, frequency, waveform, exposure duration, treatment regimens, and coil or magnet configurations. The diversity of assessed endpoints, such as proliferation, apoptosis, oxidative stress, gene and protein expression, metabolic activity, and tumor growth impedes cross-study comparability and interferes with quantitative synthesis. The MS protocol heterogeneity restricts the direct interpretation of results and the determination of a standardized protocol. In addition, the studies showed that the efficacy of MS is dependent on the nature and location of the cancer and the cell type and metabolic state. The studies analyzed in the current review include numerous limitations that affect the clarity and usefulness of data, such as the lack of adequate methodological explanations, exhibiting a moderate to high risk of bias related to sample selection, blinding, randomization, and outcome reporting. These barriers collectively reduce reproducibility and significantly restrict the translational interpretation of current evidence, making it premature to draw major mechanistic or clinically applicable conclusions from the existing preliminary findings.

## 5. Conclusions and Final Considerations

This scoping review mapped the current knowledge on MS’s effects on tumor biology across experimental models and stimulation protocols. The findings show that both rMS and sMS elicit a diverse array of biological responses in cancer cells, which are very variable and greatly influenced by tumor type, stimulation frequency, magnetic field intensity, exposure length, and experimental conditions. A common subject in the literature is MS as an adjunct to traditional anticancer treatments. Evidence suggests that both rMS and sMS may enhance drug delivery, increase intracellular drug retention, disrupt tumor cell membranes, modulate drug resistance pathways, and selectively sensitize cancer cells to chemotherapeutic agents, including in brain tumor models through modulation of the blood–brain barrier. Although intriguing, these data are preclinical and methodologically varied. The lack of defined stimulation procedures, comparison studies across tumor types, and integration of functional, molecular, and translational outcomes are major knowledge gaps. Future research should prioritize systematic parameter optimization, standardized reporting frameworks, and well-designed in vivo and translational studies to clarify the conditions under which MS may yield reproducible and clinically relevant effects.

## Data Availability

The raw data supporting the conclusions of this article will be made available by the authors on request.

## Supplementary Materials

The following supporting information can be downloaded at: https://www.mdpi.com/article/10.3390/biomedicines14030638/s1, Supplementary File S1: Preferred Reporting Items for Systematic reviews and Meta-Analyses extension for Scoping Reviews (PRISMA-ScR) checklist; Supplementary File S2: Search syntax.

## Abbreviations

The following abbreviations are used in this manuscript:

ATP	Adenosine Triphosphate
bRMS	Burst Repetitive Magnetic Stimulation
BDNF	Brain-Derived Neurotrophic Factor
CCK-8	Cell Counting Kit 8
CFA	Colony Formation Assay
CHX	Cycloheximide
CNS	Central Nervous System
CRC	Colorectal Cancer
cTBS	Continuous Theta Burst Stimulation
DCFDA	2′,7′-Dichlorofluorescin Diacetate
EMT	Epithelial–Mesenchymal Transition
FAAS	Flame Atomic Absorption Spectrophotometer
Fe^2+^	Ferrous Iron
FGF-2	Fibroblast Growth Factor 2
FITC	Fluorescein Isothiocyanate
GDNF	Glial Cell Line-Derived Neurotrophic Factor
GO	Gene Ontology
H&E	Hematoxylin–Eosin
H’ase III	Heparin Lyase III
HCC	Hepatocellular Carcinoma
HPLC	High-Performance Liquid Chromatography
HS	Heparan Sulfate
IF	Immunofluorescence
IGF-Trap	IGF Signaling Inhibitor
IHC	Immunohistochemistry
IL-2	Interleukin-2
iRMS	Intermittent Repetitive Magnetic Stimulation
iTBS	Intermittent Theta Burst Stimulation
LLC	Lewis Lung Carcinoma
LTD	Long-Term Depression
LTP	Long-Term Potentiation
MDA	Malondialdehyde
MMP-2	Matrix Metalloproteinase-2
MTT	3-(4,5-Dimethylthiazol-2-Yl)-2,5-Diphenyl-2H-Tetrazolium Bromide
NIBS	Non-Invasive Brain Stimulation
NSS	Neurological Severity Score
NT-3	Neurotrophin-3
PD	Platycodin D
PDGF	Platelet-Derived Growth Factor
PI	Propidium Iodide
PMC	Puromycin
RNA-seq	RNA Sequencing
rMS	Repetitive Magnetic Stimulation
ROS	Reactive Oxygen Species
RT-qPCR	Reverse-Transcriptase Quantitative Polymerase Chain Reaction
rTMS	Repetitive Transcranial Magnetic Stimulation
SEM	Scanning Electron Microscope
siRNA	Small Interfering RNA Transfection
SMF	Static Magnetic Field
sMS	Static Magnetic Stimulation
TEM	Transmission Electron Microscope
TBS	Theta Burst Stimulation
TGF-β1	Transforming Growth Factor β1
TMS	Transcranial Magnetic Stimulation
TMZ	Temozolomide
TNF-α	Tumor Necrosis Factor Alpha
TUNEL	Terminal dUTP Nick-End Labeling Assay
WM2	Water-Cooled Magnet
