# Supplementary material for: Molecular and Cellular Mechanisms of Static and Repetitive Magnetic Stimulation in Cancer Therapy: A Scoping Review
Source: Biomedicines. 2026 Mar 12;14(3):638. doi: 10.3390/biomedicines14030638 (PMC13024157; doi:10.3390/biomedicines14030638)
Supplement: Supplementary file 1 [file biomedicines-14-00638-s001.zip › Supplementary File S2 - Search strategy.pdf]

Supplementary File 2 - Search Syntax

PUBMED / MEDLINE

- ("magnetic stimulation"[Title/Abstract] OR "repetitive magnetic stimulation"[Title/Abstract] OR "pulsed magnetic stimulation"[Title/Abstract]) AND ("cancer"[Title/Abstract] OR "neoplasm"[Title/Abstract] OR "tumor"[Title/Abstract]) AND ("cells"[Title/Abstract] OR "cell line"[Title/Abstract] OR "rodent"[Title/Abstract])
- ("magnetic stimulation"[Title/Abstract] OR "static magnetic stimulation"[Title/Abstract]) AND ("cancer"[Title/Abstract] OR "neoplasm"[Title/Abstract] OR "tumor"[Title/Abstract]) AND ("cells"[Title/Abstract] OR "cell line"[Title/Abstract] OR "rodent"[Title/Abstract])

EMBASE

- ("magnetic stimulation" OR "repetitive magnetic stimulation" OR "pulsed magnetic stimulation") AND (cancer OR neoplasm OR tumor) AND (cells OR "cell line" OR rodent)
- ("magnetic stimulation" OR "static magnetic stimulation") AND (cancer OR neoplasm OR tumor) AND (cells OR "cell line" OR rodent)

WEB OF SCIENCE

- TS= (("magnetic stimulation" OR "repetitive magnetic stimulation" OR "pulsed magnetic stimulation") AND (cancer OR neoplasm OR tumor) AND (cells OR "cell line" OR rodent))
- TS= (("magnetic stimulation" OR "static magnetic stimulation" AND (cancer OR neoplasm OR tumor) AND (cells OR "cell line" OR rodent))

SCOPUS

TITLE-ABS-KEY

- (("magnetic stimulation" OR "repetitive magnetic stimulation" OR "pulsed magnetic stimulation") AND (cancer OR neoplasm OR tumor) AND (cells OR "cell line" OR rodent))
- (("magnetic stimulation" OR "static magnetic stimulation") AND (cancer OR neoplasm OR tumor) AND (cells OR "cell line" OR rodent))
